# Optimization of a Microwave-Coupled Enzymatic Digestion Process to Prepare Peanut Peptides 

**DOI:** 10.3390/molecules17055661

**Published:** 2012-05-11

**Authors:** Huicui Zhang, Lina Yu, Qingli Yang, Jie Sun, Jie Bi, Shaofang Liu, Chushu Zhang, Lin Tang

**Affiliations:** 1 Shandong Normal University, Jingshi Road No. 167, Lixia District, Jinan 250014, China; Email: dacui1201@163.com (H.Z.); tang_lin@21.com.cn (L.T.); 2 Shandong Peanut Research Institute, Wannianquan Road No. 126, Licang District, Qingdao 266100, China; Email: lhtyln0626@yahoo.com.cn (L.Y.); sj605@sina.com (J.S.); bj.baby@163.com (J.B.);lsf909@sina.com (S.L.); zcs.2003@163.com (C.Z.)

**Keywords:** microwave, peanut peptides, peanut protein, response surface methodology

## Abstract

The best enzyme to prepare peanut peptides, papain, coupled with microwave irradiation was selected from five common proteases according to the results of the yield of peanut peptides [nitrogen solution index (NSI) in trichloroacetic acid (TCA), TCA-NSI] and the degree of hydrolysis (DH). The main factors that influenced the microwave-coupled enzymatic digestion method were optimized by response surface analysis. The optimal conditions obtained were as follows: microwave extraction time, 9.5 min; power, 600 W; substrate concentration, 4%; enzymatic reaction temperature, 50 °C; enzyme quantity, 6,500 U/g; pH, 7.1 (phosphate buffer, 0.05 mol/L). Under these conditions, TCA-NSI was 62.00% and DH was 25.89%, which is higher than that obtained with either protease hydrolysis or microwave hydrolysis alone.

## 1. Introduction

Peanuts are the World's fourth most important source of edible vegetable oil and the third most important source of vegetable protein feed meal [1]. In China, peanuts have been grown as an oil seed crop for export, producing the edible oil, whereas the protein residue in the form of oil cake can be used for animal feed [2,3]. Recently peanut protein has been receiving increasing attention from the food industry, as an additive in meat and dairy products, baked foods, health/functional foods and other similar commercially important items. One of the notable features of peanut protein is its high nutritional value, although its functional properties, digestibility and bioactivity are relatively low [4]. Its hydrolysate, however, a peanut peptide, could have better physicochemical properties such as solubility, emulsifying capacity, foam capacity, *etc*. Certain peptide sequences that are correlated with potent antioxidant and radical scavenging functions have been identified by sequence comparison of various proteins and are known to be present in peanut protein. The antioxidant activity of antioxidant peptides can be improved if Pro, Tyr, and His are in suitable sites in the peptide chain. The dipeptide with Met and His being in C-terminal has higher antioxidant activity, while Trp being in N-terminal has higher antioxidant activity [5]. Peptides containing His have antioxidant effects through chelation of transition metal ions. His molecules have α-amino groups, carboxyl groups, and imidazolyl active side chain groups. The main chelating mechanism is that the α-amino group, carboxyl group and metal ion can give rise to a five ring; α-amino group, imidazolyl and metal ion can form a six ring; and carboxyl group, imidazolyl and metal ion can produce a seven ring [6]. Besides, the interaction of the side chain carboxyl group of acidic amino acids in peptides and metal ions can passivate metal ion oxidation, and thus weaken free radical chain reactions and display antioxidant functions [7]. Therefore, peanut peptides will have important potential applications due to their physiological function, such as antioxidant activities, scavenging of free radicals, angiotensin-converting enzyme (ACE) inhibitory activity.

Peptides can be obtained from either chemical or enzymatic hydrolysis of proteins, but enzymatic hydrolysis is generally preferred from the food safety point of view [8]. This preference can be attributed to the fact that chemical hydrolysis can destroy L-amino acids, produce D-amino acids, and form toxic substances such as lysinoalanine and also because enzymatic hydrolysis is moderately cheaper, more specific, and less destructive than chemical hydrolysis, which ultimately destroys all peptide bonds [9,10,11,12,13]. Microwave radiation can, through the solvent, reach the inner dissolved materials directly. This heating method is fast and uniform throughout the material, making it faster and more effective than traditional heating methods [14,15]. To simulate biological systems and enhance the yield of functional peptides, microwave-coupled enzymatic reactions are carried out under optimal conditions. The degree of hydrolysis, a measure of protein degradation, is a controlling parameter for the process. It also serves as a means of determining protein hydrolysate properties. In this paper, a microwave-coupled enzyme method was introduced to prepare peanut peptides from isolated peanut protein. Degree of hydrolysis (DH) was taken as response variable, and the four factors (including microwave time, microwave temperature, pH, and enzyme concentration) were selected to conduct a response surface analysis (RSA).

## 2. Results and Discussion

### 2.1. Selection of the Optimal Enzyme

**Figure 1:**
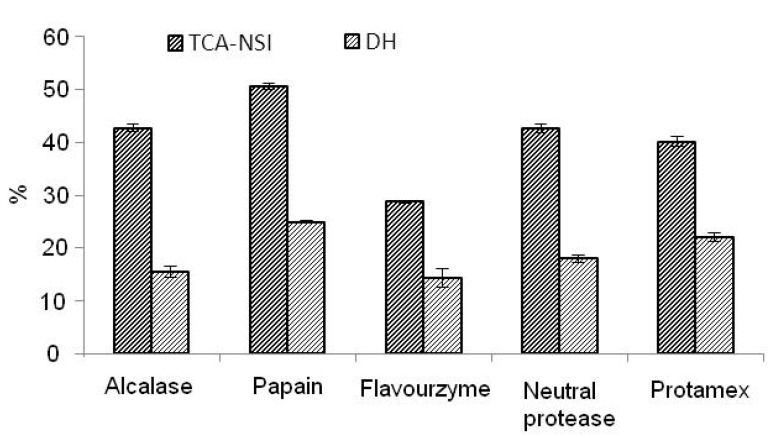
Selection of proteases.

Many studies have shown that the physicochemical and antioxidant properties of peanut protein hydrolysate were positively related with the degree of hydrolysis. The five kinds of familiar and industrial production proteases, Alcalase, papain, Flavourzyme, neutral protease, and Protamex were chosen as the reaction enzymes. As shown in Figure 1, it was found that papain was the best enzyme and it was thus selected for microwave-coupled hydrolysis of peanut protein isolate according to the results of the higher yield of peanut peptides (TCA-NSI) and the higher degree of hydrolysis (DH).

### 2.2. Effect of the Single Factor Test

#### 2.2.1. Effect of Microwave Power on TCA-NSI and DH

**Figure 2:**
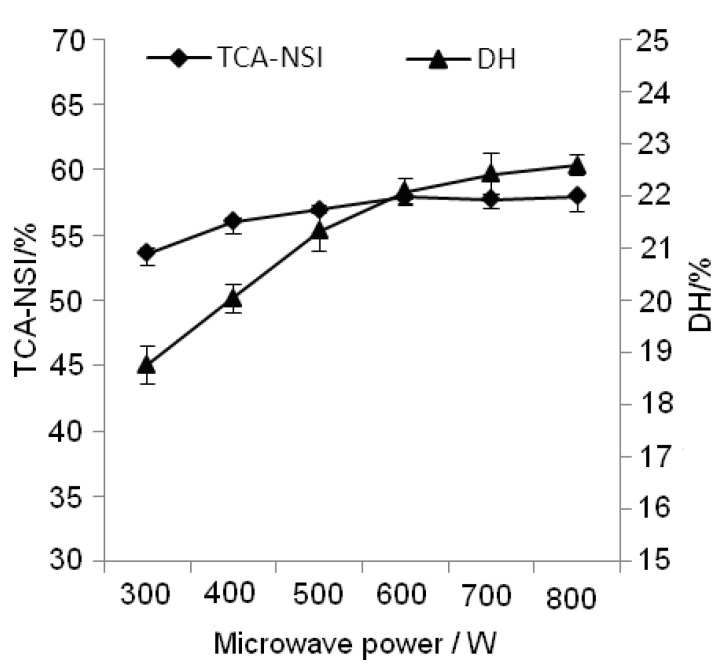
Effect of microwave power on TCA-NSI and DH.

The effects of different microwave powers on TCA-NSI and DH were studied using the single factor methodology. Figure 2 shows that TCA-NSI and DH increased with increasing microwave power. 

The hydrolysate temperature rise could be accelerated under the high-power microwave condition. However, when the microwave power reached 600 W, the TCA-NSI and DH were close to the maximum and little significant improvement was seen above 600 W. Additionally the higher microwave power represents a greater energy consumption, so in terms of economizing on energy, 600 W of microwave power was the most appropriate choice.

#### 2.2.2. Effect of Microwave Time on TCA-NSI and DH

**Figure 3:**
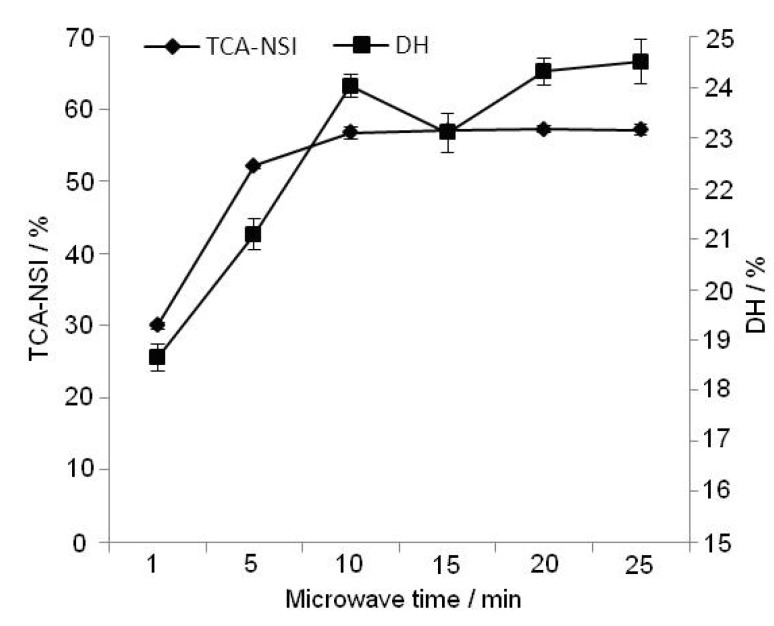
Effect of microwave time on TCA-NSI and DH.

Microwave radiation can penetrate through the solvent to reach the interior of materials directly. Therefore, irradiation can allow for a shorter reaction time than enzymatic hydrolysis alone. Hydrolysis time was an important basis for the choice of different hydrolysis methods. The effects of different microwave times on the extraction rate of peanut protein isolate were studied using the single factor methodology. The reaction conditions were microwave power 600 W, microwave time 1, 5, 10, 15, 20, 25 min, temperature 50 °C, pH 7.0, enzyme dosage 6,000 U/g, substrate concentration 4%. In the initial hydrolysis event, the concentration and the enzymatic activity is high, so the protein was hydrolyzed quickly. Figure 3 shows that with increasing irradiation times, both TCA-NSI and DH increased. They were close to the maximum values when the hydrolysis time was 10 min. As the hydrolysis progressed, the enzymatic activity was reduced and the substrate concentration was low. If the hydrolysis time was extended further, no obvious increase of TCA-NSI and DH was noted. Both protease structure and substrate protein structure can be influenced on microwave energy. The enzyme will become inactive if the microwave treatment time is too long (more than 15 min). The shorter hydrolysis time had little influence on the extraction, which was mainly achieved by the hydrolysis process. The production cycle was also decreased with shorter hydrolysis times. Therefore, 5 min~15 min was selected as the best time in the RSM experiments.

#### 2.2.3. Effect of Microwave Temperature on TCA-NSI and DH

**Figure 4:**
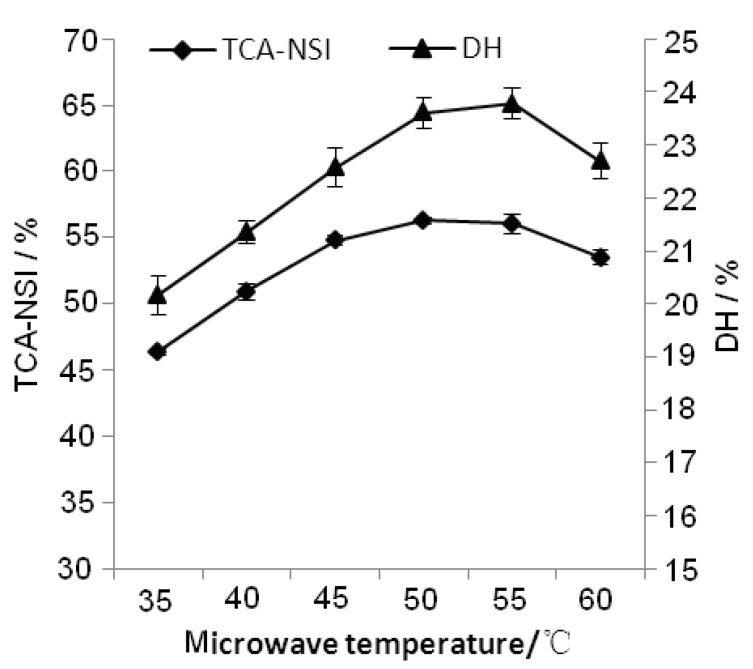
Effect of microwave temperature on TCA-NSI and DH.

The effects of different microwave temperatures on TCA-NSI and DH were studied using the single factor methodology. Figure 4 shows that initially, with increasing temperature, TCA-NSI and DH continued to rise. After the temperature reached 50 °C, they began to show a downtrend. The stability of the enzyme was closely related to the hydrolysis temperature. This is because that the protease has a specific spatial structure, and would be irreversible denatured if the reaction temperature was too high, and then the enzymatic activity would be lost or decreased, whereas when the temperature was too low, the random molecular motion is not more vigorous than at a high temperature, the protease has a lower probability of collision. Consequently the temperature in the hydrolysis process should not be too high or too low. Therefore, an optimum temperature range of 45 °C to 55 °C was selected for the final test hydrolysis of peanut protein isolate.

#### 2.2.4. Effect of pH on TCA-NSI and DH

The influence of pH is mainly manifested in the activity of the enzyme. The specific spatial structures of the enzyme would be broken and the conformational changed under conditions of acidity (low pH) or alkalinity (high pH). The initial pH value of every reaction solution can be controlled with different phosphate buffers (0.05 mol/L). 

**Figure 5:**
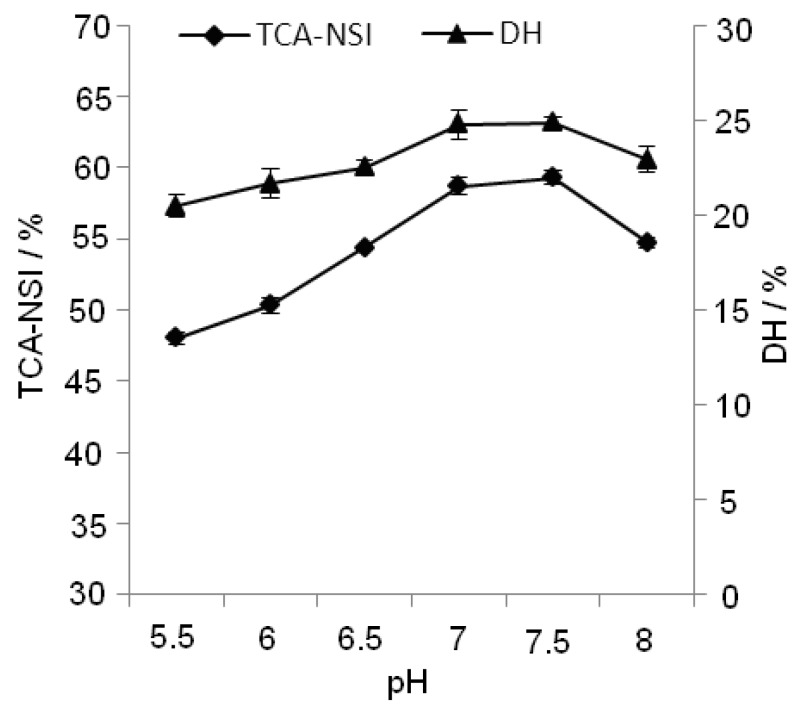
Effect of pH on TCA-NSI and DH.

The optimum pH of the papain is 6.5, which was obtained from the manufacturer, but as the reaction progressed, the acidity of the system would increase. Figure 5 shows that TCA-NSI and DH increased with increasing pH. When the pH reached optimum value (6.5), TCA-NSI and DH were high, but they showed a downward trend when the pH was above 7.5. Because of the alkalinity of the system, the enzymatic activity was reduced. Therefore, we chose pH range of 6.5 to 7.5 as the pH value in the RSM experiment.

#### 2.2.5. Effect of Enzyme Dosage on TCA-NSI and DH

**Figure 6:**
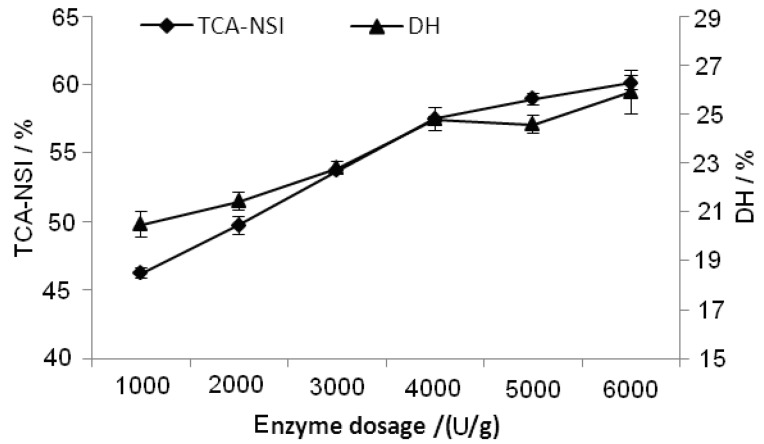
Effect of enzyme dosage on TCA-NSI and DH.

During enzymatic hydrolysis, the amount of peanut protein isolate decreased while the amounts of peptides increased. With increasing enzyme dosage, protein hydrolysis proceeded more quickly to form peptides. At very high enzyme dosage, the initial velocity of reaction would be increased but the degree of the hydrolysis would not be changed because the amount of protein available would be the limiting factor. The experimental results (Figure 6) showed that higher enzyme dosage increased the TCA-NSI and DH. When the enzyme dosage reached 6,000 U/g, increases of TCA-NSI and DH were modest. Therefore, we chose 4,000~6,000 U/g as the optimum enzyme dosage.

#### 2.2.6. Effect of Substrate Concentration on TCA-NSI and DH

**Figure 7:**
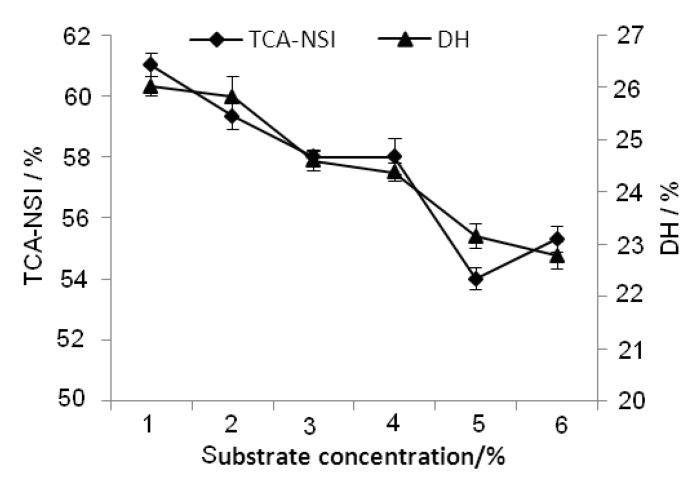
Effect of substrate concentration on TCA-NSI and DH.

The experimental results (Figure 7) showed that TCA-NSI and DH decreased with increasing substrate concentration. Technically a high substrate concentration will reduce the availability of water in the reaction system and the diffusion motions of protease and the substrate becomes aggregated. Hence, the hydrolysis was inhibited. However, when the concentration of substrate was too low, the probability of collisions between the substrate and protease will be decreased and the hydrolysis would also be inhibited. According to the experimental results, the high concentration inhibition was more obvious than low concentration inhibition in the range of substrate concentrations (1%~6%) tested. However, in practical production, if the substrate concentration was low, there would be more energy dissipated per unit of product produced and the utilization of equipment would not be optimal. Therefore, we chose 4% as the optimum substrate concentration.

### 2.3. Optimization of the Process

#### 2.3.1. Response Surface Analysis Plan and Analysis of Results

**Figure 8:**
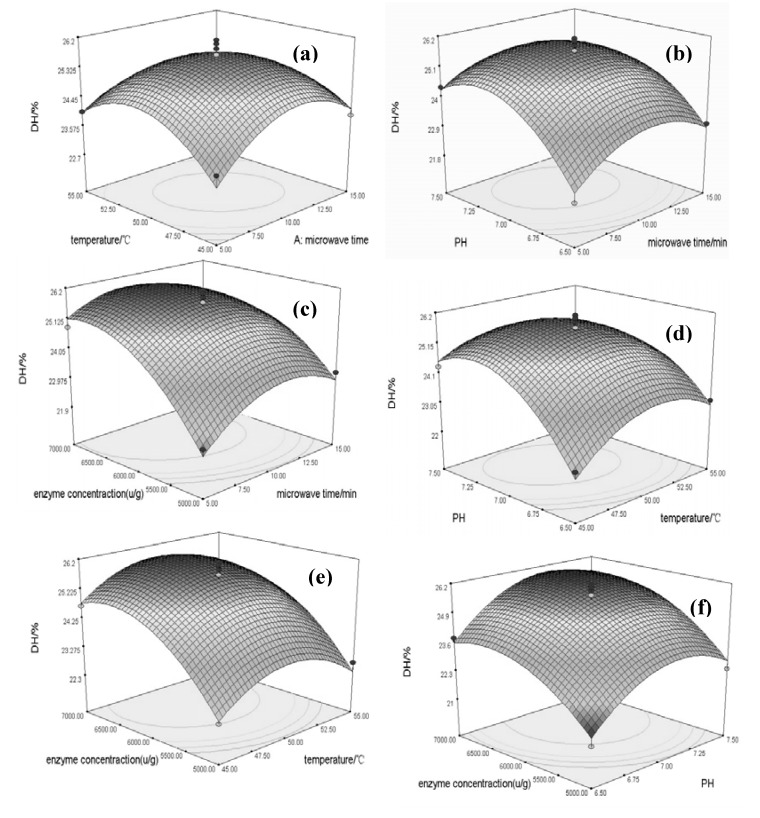
Response surfaces showing the interactive effects of different factors on DH (**a**) microwave time and temperature; (**b**) microwave time and pH; (**c**) enzyme concentration and time; (**d**) pH and temperature; (**e**) enzyme concentration and temperature; (**f**) enzyme concentration and pH).

The design matrix of the variables in the coded units is shown in Table 1, along with the predicted and experimental response values for DH. The predicted values of responses (COD yield) were obtained by quadratic model fitting techniques using the Design Expert software. The statistical model was developed by applying multiple regression analysis methods using the experimental data for the DH in the treatment, which can be given as:


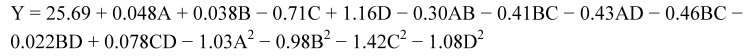


where Y is the DH(%); A is the microwave time(min); B is the temperature (°C); C is the pH and D is the enzyme concentration (U/g). The model was checked using the F-test, and the analysis of variance (ANOVA) for the response surface quadratic model is summarized in Table 2. In Table 2, the Model F-value of 21.73 implies that the model is significant. There is only a 0.01% chance that a Model F-value this large could occur due to noise. There is a very low probability value (P model, F < 0.0001). Values of “Prob > F’’ less than 0.0500 indicate that model terms are significant. Further evidence is provided by the lack-of-fit F-value. The lack-of-fit F-value of 0.37 implies the lack of fit is not significant relative to the pure error. There is a 90.82% chance that a lack-of-lit F-value this large could occur due to noise. A non-significant lack of fit is good and, in this case, all the model coefficients, namely C, D, AD, BC, A^2^, B^2^, C^2^, D^2^, are significant (Table 2). The goodness of the model can be checked by determining the coefficient R^2^ and the adjusted R^2^ (multiple correlation coefficient R). The value of adjusted R^2^ (0.9120) for Eq. (1) suggests that the total variation of 91.20% for DH can be attributed to the independent variables and only about 8.80% of the total variation cannot be explained by the model. The closer the values of adjusted R^2^ are to 1, the better is the correlation between the experimental and predicted values [16,17]. Here, the predicted R^2^ of 0.8426 is in reasonable agreement with the adjusted R^2^ of 0.9120 between the experimental and predicted values of DH. “Adeq precision” measures the signal-to-noise ratio. A ratio greater than 4 is desirable. The ratio of 15.190 for the Model indicates an adequate signal (Table 2). This model can be used to navigate the design space. The fitted response surface plot was generated by the statistically significant above model using the Design Expert program to understand the interaction of the parameters required for optimum DH. The plots shown in Figure 8 were then used to facilitate plotting of three-dimensional surface and contour plots. Two parameters of each model were plotted at any one time on the X and Y axes with the yield on the Z axis. The 3D plots were drawn directly to illustrate the main and interactive effects of the independent variables on the dependent variables and the response surface, whose coefficients were given in Table 2, is shown in Figure 8. We found the optimum process parameters and the interactions among parameters from the culmination and contour of the response surface graph. We could see the existence of an extremum in the selected area; the culmination of the response surface was also the central point of the minimum ellipse of the contour line.

**Table 1:** Arrangement of four-variable, three-level response surface central composite design and experimental data of DH.

Serial number	A	B	C	D	DH/%
1	−1	−1	0	0	23.48
2	1	−1	0	0	23.91
3	−1	1	0	0	24.01
4	1	1	0	0	22.75
5	0	0	−1	−1	21.07
6	0	0	1	−1	22.41
7	0	0	−1	1	23.79
8	0	0	1	1	26.07
9	−1	0	0	−1	22.17
10	1	0	0	−1	23.19
11	−1	0	0	1	24.04
12	1	0	0	1	24.10
13	0	−1	−1	0	22.31
14	0	1	−1	0	23.14
15	0	−1	1	0	24.34
16	0	1	1	0	21.17
17	−1	0	−1	0	21.90
18	1	0	−1	0	23.02
19	−1	0	1	0	24.37
20	1	0	1	0	25.65
21	0	−1	0	−1	22.31
22	0	1	0	−1	22.74
23	0	−1	0	1	24.67
24	0	1	0	1	25.01
25	0	0	0	0	25.87
26	0	0	0	0	26.10
27	0	0	0	0	26.01
28	0	0	0	0	24.79
29	0	0	0	0	25.69

**Table 2:** Analysis of variance (ANOVA) for the quadratic regression model for extraction yield of peanut protein isolate from defatted peanut powder as a function of microwave power, microwave extraction time, microwave extraction temperature and solid-to-liquid ratio.

Source	Sum of squares	df	Mean square	F value	*p*-value Prob > F
Model	46.85	14	3.35	21.73	<0.0001
A	0.028	1	0.028	0.18	0.6761
B	0.018	1	0.018	0.11	0.7401
C	6.05	1	6.05	39.28	<0.0001
D	16.19	1	16.19	105.14	<0.0001
AB	0.35	1	0.35	2.30	0.1517
AC	0.66	1	0.66	4.31	0.0567
AD	0.76	1	0.76	4.91	0.0437
BC	0.85	1	0.85	5.50	0.0343
BD	2.025E-003	1	2.025E-003	0.013	0.9103
CD	0.024	1	0.024	0.16	0.6998
A^2^	6.89	1	6.89	44.71	<0.0001
B^2^	6.20	1	6.20	40.27	<0.0001
C^2^	13.04	1	13.04	84.66	<0.0001
D^2^	7.50	1	7.50	48.70	<0.0001
Residual	2.16	14	0.15		
Lack of fit	1.04	10	0.10	0.37	0.9082
Pure error	1.12	4	0.28		
Cor Total	49.00	28			

As shown in Figure 8, we can see each factor that influences the response value, in Figure 8: pH and enzyme concentration have the most significant influence on DH for the steep curve. The microwave time and temperature are not so significant, as the curves are smoother.

#### 2.3.2. Verification of Results

Using Design-expert to further analyze the experiments above, we find that the best extraction conditions are: microwave time 9.63 min, pH 7.14, temperature 49.60 °C, enzyme concentration 6509.50 U/g; under these conditions, DH could reach 26.12%. Considering the convenience of practical operations, we revised the process parameters to: microwave time 9.5 min, pH 7.1, temperature 50 °C, enzyme concentration 6,500 U/g. Three parallel tests were carried out to verify the validity of the experiment; the results for the protein extraction rate are 25.98%, 26.07%, and 25.90%; the average of the results is 25.98%. The results indicate that the theoretical analysis is a good match for the experimental results. This indicates that the optimization design method is feasible based on the response surface analysis.

## 3. Experimental

### 3.1. Materials

Defatted peanut protein powder was purchased from Shandong Tianshen Biological Protein Co., Ltd. (Linyi, China). Other reagents included: disodium tetraborate decahydrate, Na-dodecyl-sulfate (SDS), *o*-phthaldialdehyde 97% (OPA), ethanol, dithiothreitol 99% (DTT), serine, trichloroacetic acid (TCA) (all produced by Sinopharm Chemical Reagent Co., Ltd., Shanghai, China), Alcalase (Novozymes A/S), papain (Nanning Panglong; China), Protamex (Novozymes A/S), neutral protease (Nanning Panglong; China), flavourzyme (Novozymes A/S).

### 3.2. Experimental Analysis

#### 3.2.1. Protease Activity Measurements

The Folin method was used to measure the protease activity [18]. The peptide recovery ratio (TCA-NSI) was estimated by measuring the nitrogen content of hydrolyzed proteins solubilized in 10% trichloroacetic acid. 10 mL of enzymatic hydrolysates were mixed with 10 mL of 20% TCA and then centrifuged at 4,000 r/min for 15 min [19]. The peptide recovery ratio was calculated according to: 


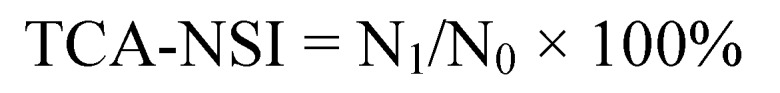


where N_1_ is the nitrogen content (in mg) soluble in 10% trichloroacetic acid and N_0_ is the total nitrogen (in mg).

#### 3.2.2. Degree of Hydrolysis (DH) of the Protein Hydrolyses

The spectrophotometric OPA method was used to analyze the DH, as follows [20]:


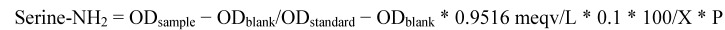


where serine-NH2 = meqv serine NH2/g protein; X = g sample; P = protein % in sample; 0.1 is the sample volume in liter (L). The value of h is then: 


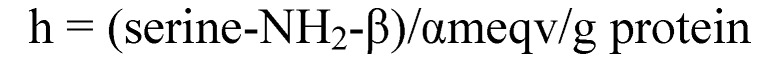


where α = 1, β = 0.4. DH is calculated: 


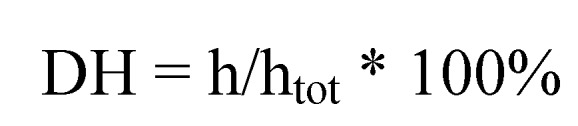


where _htot_ = 7.13.

### 3.3. Peanut Protein Isolate Preparation Process

The peanut protein isolate can be prepared by the method of alkali-dissolution and acid sedimentation. Defatted peanut protein powder was added to NaOH solution (pH 9.0, 0.01 mmol/L) to form an alkali-soluble homogeneous solution, and this was placed in the microwave extraction apparatus. The sample was extracted two times at constant microwave power and temperature for a certain time. After the reaction was complete, the reaction mixture was centrifuged. The supernatant was added to hydrochloric acid to adjust pH 4.5 in order to obtain the isoelectric point precipitation protein. When the pH value was 4.5, large amount of white precipitate were formed. This shows that the isoelectric point precipitation reaction has been completed. Then, the mixture was centrifuged and the precipitate was freeze-dried in order to obtain peanut protein isolated.

### 3.4. Preparation of Peanut Peptides

The experiments were conducted in a 200-mL Erlenmeyer flasks containing 4 g peanut protein isolate and 100 mL distilled water. The flasks were heated in a water bath (90 °C; B-260, Shanghai Yarong, China) to inactivate the protein. The initial pH of the protein sample was obtained using different phosphate buffer (0.05 mol/L). An amount of enzyme was added to the sample. Then the sample was transferred into the microwave (XH-100A; Beijing Xianghu, China) for hydrolysis. When the reaction was finished, the sample was heated in a water bath at 100 °C to inactivate the enzyme. Finally, the degree of hydrolysis was determined by the OPA method.

### 3.5. Selection of the Optimal Enzyme

The enzymes most commonly used to hydrolyze peanut protein are Alcalase, Flavourzyme, Protamex, neutral protease, and papain. The DH and TCA-NSI were measured under optimal reaction conditions for Alcalase (pH 8.0, T 55 °C, 1,214 U/g, 15 min), Flavourzyme (pH 7.0, T 55 °C, 739 U/g, 15 min), Protamex (pH 7.0, T 55 °C, 1,188 U/g, 15 min), neutral protease (pH 6.0, T 55 °C, 1,200 U/g, 15 min), and papain (pH 6.5, T 55 °C, 2,000 U/g, 15 min).

### 3.6. Single Factor Design

The basic conditions of single factor design are: microwave power 400 W, microwave time 15 min, temperature 50 °C, pH 7.0, enzyme dosage 6,000 U/g, substrate concentration 4%. The pH and enzyme concentration were determined by the optimal reaction conditions of the optimal enzyme. Factors and levels of the single factor experiment were as follows: Enzyme dosage (U/g substrate): 1000, 2000, 3000, 4000, 5000, 6000; Temperature (°C): 35, 40, 45, 50, 55, 60; Time (min): 1, 5, 10, 15, 20, 25; Substrate concentration (%): 1, 2, 3, 4, 5, 6; pH: 5.5, 6.0, 6.5, 7.0, 7.5, 8.0; Microwave power (W): 300, 400, 500, 600, 700, 800.

### 3.7. Response Surface Design (RSD)

The microwave power (600 W) and substrate concentration (4%) were fixed (the effect of microwave power on protein DH was the smallest and for practical production 4% substrate concentration was the best choice) according to the principle of experimental design from the Box-Behnken Center. Combined with the results of the single factor test, we investigated the influence of microwave time (A), microwave temperature (B), pH (C), and enzyme concentration (D) on protein DH (Y). Factors: the level of experiment and the experimental design are shown in Table 1 and Table 3. The experimental design and analysis was designed using Design-Expert software.

**Table 3:** Variable and levels in four-variable, three-level response surface design.

Level	A Microwave time/min	B Microwave temperature/°C	C pH	D Enzyme concentration/(U/g)
−1	5	45	6.5	5000
0	10	50	7.0	6000
1	15	55	7.5	7000

## 4. Conclusions

In this paper the best enzyme selected from five common enzymes was coupled with microwave irradiation to prepare peanut peptides. The main factors influencing the microwave-assisted enzyme method were optimized by response surface analysis. Papain was the best enzyme of those tested. The optimal conditions were obtained as follows: the microwave extraction time is 9.5 min; the power is 600 W; the concentration of substrate is 4%; the enzymatic temperature is 50 °C; the enzyme quantity is 6,500 U/g; the pH value is 7.1. Under these conditions, a yield of peptides (TCA-NSI) of 62% was achieved, and the degree of hydrolysis (DH) was 25.89%.Comparing the microwave-assisted enzyme method with enzymatic hydrolysis only and microwave treatment only, the microwave-coupled enzymatic method requires less time and has a higher DH.
